# D-allulose ameliorates adiposity through the AMPK-SIRT1-PGC-1α pathway in HFD-induced SD rats

**DOI:** 10.29219/fnr.v65.7803

**Published:** 2021-12-21

**Authors:** Geum-Hwa Lee, Cheng Peng, Hwa-Young Lee, Seon-Ah Park, The-Hiep Hoang, Jung Hyun Kim, Soonok Sa, Go-Eun Kim, Jung-Sook Han, Han-Jung Chae

**Affiliations:** 1 Non-Clinical Evaluation Center, Biomedical Research Institute, Jeonbuk National University Hospital, Jeonju, Jeonbuk, Republic of Korea; 2 Research Institute of Clinical Medicine of Jeonbuk National University-Biomedical Research Institute of Jeonbuk National University Hospital, Jeonju, Jeonbuk, Republic of Korea; 3 Department of Oral Pathology, School of Dentistry, Jeonbuk National University, Jeonju, Jeonbuk, Republic of Korea; 4 Samyang Corp., Bundang-gu, Seongnam-si, Gyeonggi-do, Republic of Korea; 5 School of Pharmacy, Jeonbuk National University, Jeonju, Jeonbuk, Republic of Korea

**Keywords:** D-allulose, adiposity, adipose tissue, AMPK, SIRT1

## Abstract

**Background:**

Adiposity is a major health-risk factor, and D-allulose has beneficial effects on adiposity-related metabolic disturbances. However, the modes of action underlying anti-hyperglycemic and hypolipidemic activity are partly understood.

**Objective:**

This study investigated the *in vivo* and *in vitro* effects of D-allulose involved in adipogenesis and activation of the AMPK/SIRT1/PGC-1α pathway in high-fat diet (HFD)-fed rats.

**Design:**

In this study, 8-week-old male SD (Sprague Dawley) rats were divided into five groups (*n* = 8/group), (1) Control (chow diet, 3.5%); (2) 60% HFD; (3) 60% HFD supplemented with allulose powder (AP) at 0.4 g/kg; (4) 60% HFD supplemented with allulose liquid (AL) at 0.4 g/kg; (5) 60% HFD supplemented with glucose (AL) at 0.4 g/kg. All the group received the product through oral gavage for 6 weeks. Control and HFD groups were gavaged with double-distilled water.

**Results:**

Rats receiving AP and AL showed reduced body weight gain and fat accumulation in HFD-fed rats. Also, supplementation of AL/AP regulated the cytokine secretion and recovered biochemical parameters to alleviate metabolic dysfunction and hepatic injury. Additionally, AL/AP administration improved adipocyte differentiation via regulation of the PPARγ and C/EBPα signaling pathway and adipogenesis-related genes owing to the combined effect of the AMPK/SIRT1 pathway. Furthermore, AL/AP treatment mediated PGC-1α expression triggering mitochondrial genesis via activating the AMPK phosphorylation and SIRT1 deacetylation activity in adipose tissue.

**Conclusion:**

The anti-adiposity activity of D-allulose is observed on a marked alleviation in adipogenesis and AMPK/SIRT1/PGC-1α deacetylation in the adipose tissue of HFD-fed rat.

## Popular scientific summary

- D-allulose promotes deacetylation of PGC1α in adipocyte via the AMPK-SIRT1 axis
- Study provides evidence that D-allulose supplements effectively reduced high fat diet-induced adiposity by regulating metabolism in WAT.

Adiposity is advancing at an extreme velocity (1), initiated by an excess energy uptake and a deficit of energy expenditure, and involved in developing metabolic syndrome (2). Multiple defects in glucose and energy metabolism, high blood pressure, dyslipidemia, intra-abdominal accumulation, and the pro-inflammatory state define the metabolic syndrome (3). Adiposity and its associated metabolic disorders are linked to high morbidity and mortality throughout the world. Still, the cellular and molecular mechanisms that lead to the development of adiposity remain elusive.

Adipocytes possess the feature that affects metabolic homeostasis, and it is a viable therapeutic target for adiposity and diabetes (4). Abnormal lipid droplet accumulation in adipocytes is one of the factors responsible for metabolic dysfunction. Disruptions in energy homeostasis result in impaired adipocyte differentiation, leading to hyperplasia (increased number) and hypertrophy (increased size) of adipocytes (5). D-allulose, also known as D-psicose, is a C-3 epimer of D-fructose and contains a keto group. It is generally present in the commercial mixture of D-glucose, fructose, steam-treated coffee, and processed fruits. However, its availability is limited and contents in food products depend on temperature and heating time during manufacturing processes (6, 7). D-allulose is naturally available in a few plants and bacteria. Commercially available D-allulose is mainly produced either from corn or beetroot (8–11). Previous reports demonstrate that sucrose and allulose do not influence blood oxygen levels and dependent signaling or network connectivity (9). Furthermore, D-allulose alleviates metabolic disturbance and cognitive decline in prediabetic rats, inhibits intestinal α-glucosidase in rats, and induces changes in the microbial community in mice (10, 11). Moreover, D-allulose has anti-cancer and anti-tumor effects. Thus, it is unique compared to other sugars like fructose, erythritol, glucose, and sucrose (7, 12). Previous studies have indicated that only 70% of D-allulose is absorbed in small intestine and the rest is excreted through feces. The absorbed allulose excreted via urine, suggesting that D-allulose is unlikely to be stored in the body (13). D-allulose does not contribute to hepatic energy production as it is metabolized in the liver. Strikingly, sugar syrup containing D-allulose is reported to improve glucose tolerance and insulin sensitivity via hepatic glucokinase translocation relative to other sweet additives (14). Consequently, D-allulose as a zero-calorie sweetener potentially substitutes other natural sugars such as sucrose to reduce the absorption of sugar and weight gain, and also to improve insulin sensitivity and glucose metabolism in obese and diabetic patients (14).

Moreover, the mechanism underlying the anti-hyperglycemic and hypolipidemic activity of D-allulose is partly understood. In one of the *in vivo* studies, D-Allulose was quickly metabolized and excreted after the ingestion, demonstrating its influence on lipid metabolism in rats (15). AMP-activated protein kinase (AMPK) is a conservative metabolic sensor of an energy indicator during evolutionary progress in eukaryotes (16). It plays an essential role in maintaining energy homeostasis, triggering catabolic pathways that produce ATP (Adenosine triphosphate) while shutting down anabolic pathways that consume ATP upon activation of AMPK (17). Therefore, D-allulose administration contributes to the calorie deficiency and further alters the AMP/ATP ratio in the intracellular triglyceride contents that turn on the activation switch of AMPK action by phosphorylation (16). The NAD^+^-dependent type III deacetylase SIRT1 deacetylates PGC-1α, activated by AMPK-mediated increase in NAD^+^. The AMPK-induced SIRT1-mediated deacetylation of its targets explains several converging biological effects of AMPK and SIRT1 on energy metabolism (16). Interestingly, massive findings explain that SIRT1-mediated deacetylation of PGC-1α occurs during the energy demand period, such as fasting and physical activity (18). It has been identified that adiposity has an intimate relationship with the altered number or size of mature adipocytes through adipogenesis (19). Adipogenesis regulates cell differentiation that causes the conversion of preadipocytes or mesenchymal stem cells into mature adipocytes. This conversion is controlled by the sequential activation of many transcription factors (20, 21). Adipogenesis requires upregulation of various transcription factors, including the C/EBP (CCAAT (Cytidine-Cytidine-Adenosine-Adenosine)/enhancer-binding protein) family members and PPARγ (peroxisome proliferator-activated receptor γ) (22). C/EBPβ and C/EBPδ activation is induced at the early stage of differentiation, which then further activates the expression of PPARγ and C/EBPα that are recognized as vital adipogenic regulators and are considered as the central positive modulators of adipogenesis (20, 23). They together/collectively affect the induction of adipogenesis-related factors expression that continuously and interactively accomplishes adipocyte differentiation and accumulation of lipid droplets (24).

Therefore, PPARγ and C/EBPα interactively adjust each other and control the entire adipogenesis process (20). Moreover, the late differentiation stage involves the addition of lipogenesis and a diversity alteration of lipogenic genes, such as ACC (acetyl CoA carboxylase), FAS (fatty acid synthase), and aP2 (adipocyte-binding protein 2) (25). Previous studies demonstrated PPARγ as a crucial transcription regulator implicated in the generation of adipocyte differentiation (20, 26). As known, it is essential for the formation of adipocytes: a deficit of PPARγ in preadipocytes fails to differentiate into mature adipocytes. Nevertheless, other adipogenic factors are overexpressed. Additionally, other adipogenesis-related transcriptional regulators like SREBP1c (sterol regulatory element-binding protein-1c) and CREB (cyclic AMP response binding element) are necessary for converting preadipocytes into mature adipocytes during the differentiation. This conversion accelerates fatty acid metabolism by inducing the expression of PPARγ (23). Hence, PPARγ and C/EBPα are broadly considered for drug development to prevent adiposity.

Together, it can be assumed that activation of catabolic-related genes and regulation of transcription factors that downregulate or repress genes implicated in lipogenic anabolism pathways are the potential strategies to prevent adiposity. In the present study, we examined the *in vivo* and *in vitro* effects of D-allulose involved in adipogenesis and activation of the AMPK/SIRT1/PGC-1α axis in high-fat diet (HFD)-fed rats.

## Materials and methods

### D-allulose products

D-allulose powder (AP, purity, >98%) and syrup (AL, purity, >95%) were purchased from Samyang Corporation, Republic of Korea. These D-allulose products were prepared from *Microbacterium foliorum* (non-GMO).

### Culture and differentiation of 3T3-L1 preadipocytes

3T3-L1 preadipocytes were purchased from the American Type Culture Collection (ATCC). Preadipocyte cell differentiation was performed using the adipogenesis assay kit according to the manufacturer’s instructions (Cayman Chemical, USA, Item No. 10006908). Preadipocytes were maintained and cultured in Dulbecco’s modified Eagle’s medium (DMEM) supplemented with 10% fetal bovine serum (FBS) and 100 units/mL penicillin/streptomycin. The cells were cultured at 37°C in the incubator until they reached 100% confluency (0 day). Then, medium was replaced with a fresh induction medium (10% FBS, 1% penicillin/streptomycin, 5 μg/mL insulin, 1 μM dexamethasone, 0.5 mM 3-isobutyl-1-methylxanthine) in the treatment group or fresh medium alone (control group) for 3 days. Three days after induction, the medium was substituted by a differentiation medium (DM) with 5 μg/mL insulin to promote differentiation in adipocytes every day until the cells were fully differentiated for harvesting. The D-allulose treatment groups treated at concentrations of 10, 20, and 40 mM, respectively, were treated with either phosphate-buffered saline (PBS) or D-allulose for 8 days during adipogenesis. To clarify the anti-adipogenesis effect of D-allulose, D-allulose was added in the DM. Intracellular triglyceride (TG) contents were determined using a TG quantification kit according to the manufacturer’s instructions (Asan Pharmaceutical, Seoul, South Korea). Absorbance was measured by an enzyme-linked immunosorbent assay (ELISA) reader at 510 nm.

### Oil Red O staining

Intracellular lipid accumulation was measured using Oil Red O (ORO) staining. The mature 3T3-L1 adipocytes were washed with PBS and fixed with neutral formalin (10%, pH 7.4) for 10 min. After removing the formalin and washing with PBS, the fat components accumulated in the cells were sufficiently dyed with the ORO solution prepared in advance and then washed with distilled water. The lipid droplets in 3T3-L1 adipocytes were observed under a microscope. The ORO dye was extracted from stained cells using 100% isopropanol and quantified spectrophotometrically at 490 nm.

### Animal grouping and experimental protocol

40 male SD rats (8-weeks old) were purchased from Orient Science Co. (Seongnam, Korea). All the rats were housed under standard laboratory conditions (55 to 60% relative humidity, 22 ± 2°C, 12-h light/dark circadian cycle) in the laboratory animal care facilities of Jeonbuk National University Hospital. After acclimatization, rats were randomly divided into five groups: 1) Control (chow diet, Sp-10, 3.5% fat); 2) 60% HFD; 3) 60% HFD supplemented with AP at 0.4 g/kg; 4) 60% HFD supplemented with allulose liquid (AL) at 0.4 g/kg; and 5) 60% HFD supplemented with glucose (AL) at 0.4 g/kg. All the groups received the product through oral gavage for 6 weeks. Control and HFD groups were gavaged with double-distilled water. HFD groups were fed with a calorie-rich diet of 40% sucrose, 19% casein, 18% lipid (lard), 1% cholesterol, and 1% AIN-93G vitamins with the identical quantity of minerals and fiber as the control group’s diet.

### Biochemical analysis

Biochemical indicators of metabolism like alanine aminotransferases (ALT), triglycerides (TG), aspartate aminotransferase (AST), and total cholesterol (TC) levels of each group were determined using commercially available kits (Asan Pharmaceutical, Seoul, South Korea). High-density lipoprotein (HDL), low-density lipoprotein (LDL), and cholesterol were measured using kits from BioVision, Milpitas, CA, USA. Adiponectin and leptin were measured in serum using the Rat Adiponectin (ADP) ELISA kit (CUSABIO, CSB-E07271r) and the LEP ELISA kit (CUSABIO, CSB-E07433r), respectively. All the tests were performed according to the manufacturer’s instructions. The blood test (biochemical test) was analyzed according to the guidelines of the *in vitro* assay SOP (Standard Operating Procedure) of the Non-Clinical Evaluation Center of Jeonbuk National University Hospital. The rats were fasted overnight before the collection of tissue and serum.

### Histological staining

Adipose tissues were fixed with 4% formaldehyde and embedded in paraffin and sectioned at 4-µm. For histological examination, slides were deparaffinized with xylene, and rehydrated using an alcohol gradient before staining with H&E (Hematoxylin and Eosin). To measure the adipocyte size, eight rats per group were used, and the diameter of 100 cells from four sections per group was analyzed using ImageJ software (National Institutes of Health, Bethesda, MD).

### Western blotting

3T3-L1 adipocytes and adipose tissue are lysed with RIPA lysate. Specifically, adipose tissue is homogenized on ice using a homogenizer with three times the lysate, followed by centrifugation at 14,000 rpm for 10 min at room temperature. Protein samples were separated by 4–12% sodium dodecyl sulfate-polyacrylamide gel electrophoresis and transferred to a PVDF (Polyvinylidene difluoride) membrane. After blockade of non-specific protein binding with 5% BSA (Bovine serum albumin) or 5% non-fat dry milk, membranes were probed with primary antibodies against anti-PPARγ, anti-C/EBP1α, anti-aP2, anti-FAS, anti-SERBP1c, P-AMPK, AMPK (Cell Signaling Technology, Inc., Danvers, MA, USA), and SIRT1, PGC-1α, and β-actin (Santa Cruz Biotechnology, CA, USA). Membranes were visualized using the enhanced chemiluminescence (ECL) system (Bio-Rad, Hercules, CA, USA), and band intensity was quantified using ImageJ software (National Institutes of Health, Bethesda, MD).

### Protein acetylation measurements and sirtuin activity

Immunoprecipitation was performed to isolate PGC1α and quantify its acetylation status selectively. Protein concentrations in samples were assessed by the Bradford method using lysates. In total, 500 µg of protein was added to 2 µg of rabbit IgG control and 20 µL of protein agarose for preclearance, blended at 4°C for 1 h, and then centrifuged at 12,000 g for 10 min at 4°C. The supernatant is collected and incubated overnight at 4°C with 10 µL of PGC1α immunoprecipitating antibody with gentle stirring. Furthermore, 20 µL of Protein A/G-Sepharose is added and incubated in a shaking incubator at 4°C overnight. Centrifuge the immunoprecipitated mixture at 12,000 g for 15 min at 4°C, retrieve the supernatant, and store at 4°C for further analysis. For immunoblotting, immunoprecipitated proteins in the particles and the remaining proteins in the supernatants were run on prefabricated 4–15% sodium dodecyl sulfate-polyacrylamide gradient gels for electrophoresis against SIRT1 (Santa Cruz Biotechnologies, Inc., Santa Cruz, CA, USA) and anti-acetylated lysine (Cell Signaling Technologies, Inc., Danvers, MA, USA). Sirtuin deacetylase activity was measured using the fluorometric sirtuin activity assay kit (K324, BioVision, Milpitas, CA, USA) according to the manufacturer’s instructions. Fluorescence intensity at 505 nm (excitation 400 nm) was recorded and normalized to micrograms of protein.

### Reverse transcription polymerase chain reaction

Total RNA from adipose tissue and 3T3-L1 cells were isolated using Trizol solution (Invitrogen, Carlsbad, CA, USA). The isolated total RNA was quantified, and the first-strand cDNA was synthesized from 2 μg of RNA. The primers used in this study are listed in Supplementary Table 1. The reaction was carried out using the ABI 7500 Real-Time PCR system (Applied Biosystems, Foster City, CA, USA) and analyzed using ABI 7500 software. The products amplified by real-time polymerase chain reaction (PCR) were quantified using a comparative cycle threshold (Ct) method, and each sample was corrected for the amount of β-actin expression.

### Statistical analysis

All experimental data were statistically analyzed with GraphPad Prism 5.01. Data were expressed as the mean ± SEM (standard error of mean), and differences among groups were calculated using one-way analysis of variance (ANOVA) followed by *t*-test. A *P*-value of <0.05 was considered statistically significant.

## Results

### Anti-adipogenic effects of D-allulose in 3T3-L1 cells

**Fig. 1:**
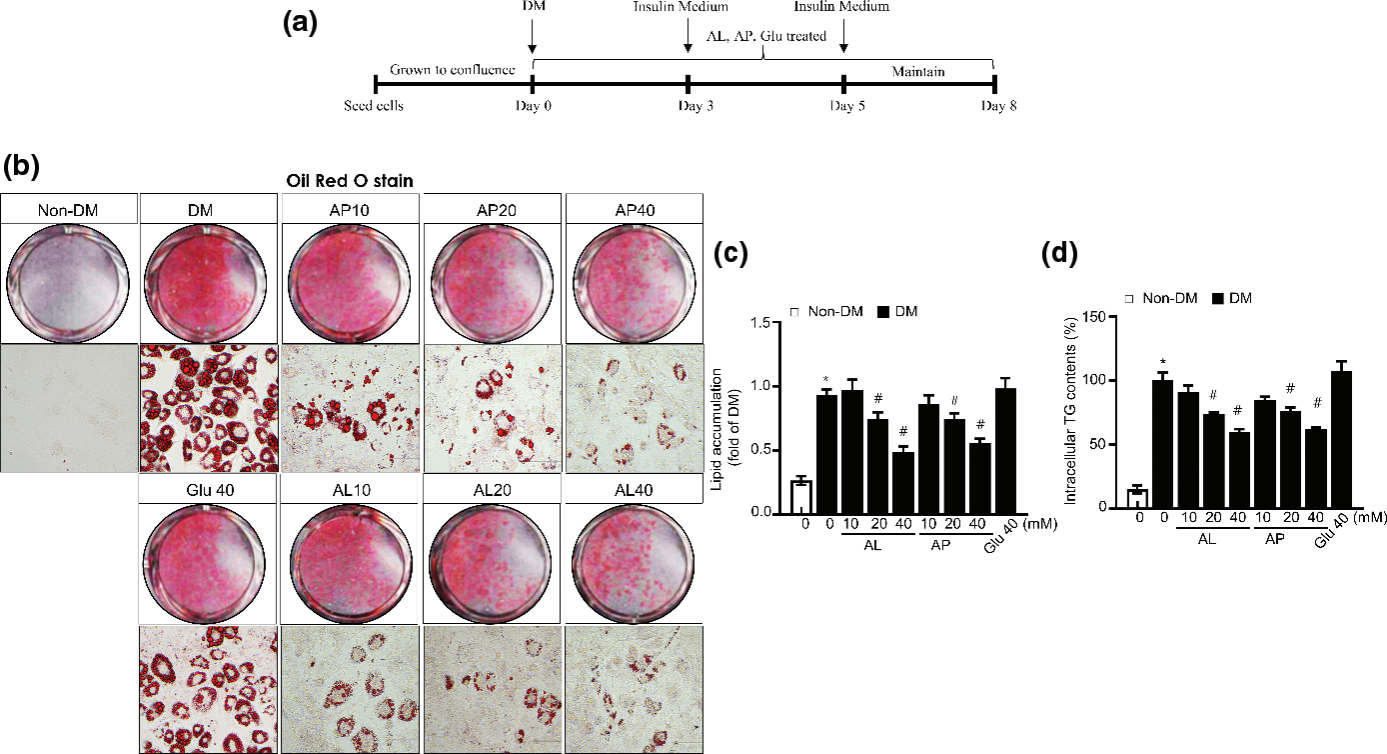
Anti-adipogenic effects of D-allulose in 3T3-L1 adipocytes. (a) Schematic diagram of the differentiation protocol for 3T3-L1 differentiation. (b, c) Effects of D-allulose on the differentiation of 3T3-L1 cells. 3T3-L1 cells were cultured in adipocyte differentiation cocktail media with or without the treatment of D-allulose. After 7 days of culture, the cells were stained with ORO and then photographed under the microscope (magnification 40×). Measurement of relative lipid content in different treatment groups. 3T3-L1 cells were cultured in adipogenic cocktail media with or without the D-allulose treatment (Right). (d) Effect of D-allulose on the triglyceride deposition in differentiated 3T3-L1 cells. 3T3-L1 cells were cultured in adipocyte differentiation cocktail media with or without the treatment of D-allulose. Results are means ± SEM from three separate experiments. (*n* = 3, *P < 0.05 vs. Non-DM group, ^#^*P* < 0.05 vs. DM group). DM, differentiated medium; Non-DM group, undifferentiated group; DM group, differentiated group; AP, Powdery D-allulose; AL, Liquid D-allulose; Glu, glucose.

To investigate the anti-adipogenic effect in adipocytes supplemented with AL and AP, we performed a series of confirmatory tests. Figure 1a shows a schematic diagram of the study design 3T3-L1. To clarify the inhibitory effect of D-allulose on the differentiation of 3T3-L1 preadipocytes into mature adipocytes, we examined the level of differentiation and lipid accumulation using ORO staining (Fig. 1b). After treating 3T3-L1 preadipocytes with the DM, they differentiated into adipocytes and eventually triggered the marked accumulation of lipid droplets. As shown in Fig. 1c, D-allulose markedly alleviates lipid accumulation on 3T3-L1 adipocytes, displaying dose-dependent differentiation in comparison with complete differentiation cells. Conversely, the glucose-administered group did not show any effective improvement on advanced adipocyte differentiation. Triglyceride is the chief component of the lipid droplet; thus, considering TG’s importance, TG contents in cells were examined. Furthermore, high dose of D-allulose administration significantly decreased intracellular TG content compared to other groups (Fig. 1d). Also, cytotoxicity of D-allulose was not observed in all the concentrations (data not shown). Collectively, MDI (methylisobutylxanthine, dexamethasone, insulin)-induced preadipocyte differentiation decreased with supplementation of D-allulose in a dose-dependent manner.

### D-allulose alters fat accumulation-related protein expression

**Fig. 2:**
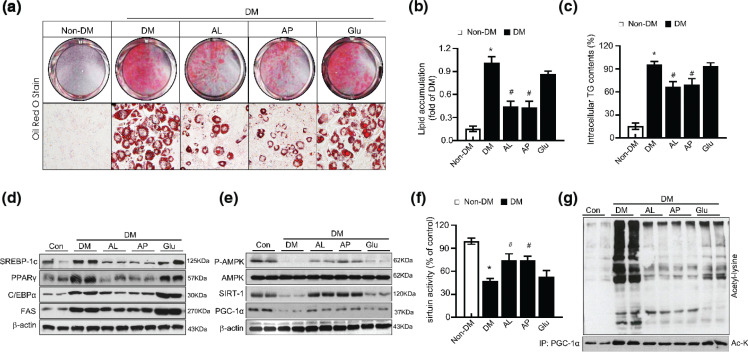
Effect of AMPK and SIRT-1 of D-allulose on deacetylation of PGC1α in 3T3-L1 adipocytes. Effects of D-allulose on the differentiation of 3T3-L1 cells. (a) Post confluent 3T3-L1 cells were differentiated in the absence or presence of 20 mM AL and 20 mM AP for 8 days. Lipid droplets were measured by ORO staining. (b) Lipid content was quantified by measuring absorbance. (c) Intracellular TG in D-allulose-treated 3T3-L1 cells was measured. (d) Expression of adipogenesis factors SREBP-1c, PPAR-γ, C/EBPα, and FAS protein levels were analyzed in 3T3-L1 cells by WB analysis. (e) Effects of D-allulose on P-AMPK, SIRT-1, and PGC-1α protein levels were analyzed in 3T3-L1 cells by WB analysis. (f) Sirtuin activity. (g) Immunoprecipitation of PGC-1α followed by WB for acetyl-lysine from differentiated 3T3-L1 cells. Results are means ± SEM from three separate experiments. (*n* = 3, *P < 0.05 vs. Non-DM group, ^#^*P* < 0.05 vs. DM-group, ^**^P < 0.05 vs. DM-Glu group). Non-DM, undifferentiated group; DM, differentiated group; AP, Powdery D-allulose; AL, Liquid D-allulose; Glu, glucose.

AP and AL treatment markedly alleviates lipid accumulation on 3T3-L1 adipocytes relative to completely differentiated cells (Fig. 2a, b). Also, allulose supplementation significantly decreased intracellular TG content compared to MDI-induced fully differentiated cells (Fig. 2c). Previous studies show that PPARγ and C/EBPα are the main transcription factors affecting adipogenesis and are abundantly expressed in adipose tissue (27). To elucidate the effect of D-allulose on the expression of crucial adipogenesis-related genes and the expression of PPARγ, C/EBPα, FAS, and SREBP-1c were analyzed via immunoblotting. Allulose supplementation markedly decreased the PPARγ and C/EBPα expression compared to completely differentiated and glucose-treated 3T3-L1 adipocytes (Fig. 2d). Further examination on the expression of adipogenic genes, such as SREBP-1c, and FAS, we observed that D-allulose treatment markedly reduced the expression of SREBP-1c and FAS relative to the differentiated cells.

### D-allulose promotes deacetylation of PGC1αin 3T3-L1 adipocyte via AMPK-SIRT1 axis

Interestingly, D-allulose treatment increased protein expression related to thermogenesis, PGC-1α in adipocytes compared to differentiated 3T3-L1 cells (Fig. 2e). Next, we investigated whether D-allulose activates the metabolic shift-associated signal transduction ‘SIRT1-AMPK signaling axis’. D-allulose recovered SIRT1, PGC-1α, and P-AMPK protein expression and SIRT1 activity in differentiated 3T3-L1 cells (Fig. 2e, f). In differentiated 3T3-L1 cells, an augment in acetyl-lysine of total protein was normalized in the D-allulose treated group (Fig. 2g). Also, it exhibited acetylation levels of PGC-1α and levels of SIRT-1 related to PGC-1α after being subjected to immunoprecipitation. SIRT-1 levels related to PGC-1α deacetylation were reduced in differentiated 3T3-L1 cells and recovered after being treated with D-allulose. D-allulose treatment in 3T3-L1 cells enhanced AMPK phosphorylation, leading to the activation of its downstream targets such as the SIRT1-PGC1α axis.

### Effect of D-allulose on mRNA expression of adipogenesis-related genes

**Fig. 3:**
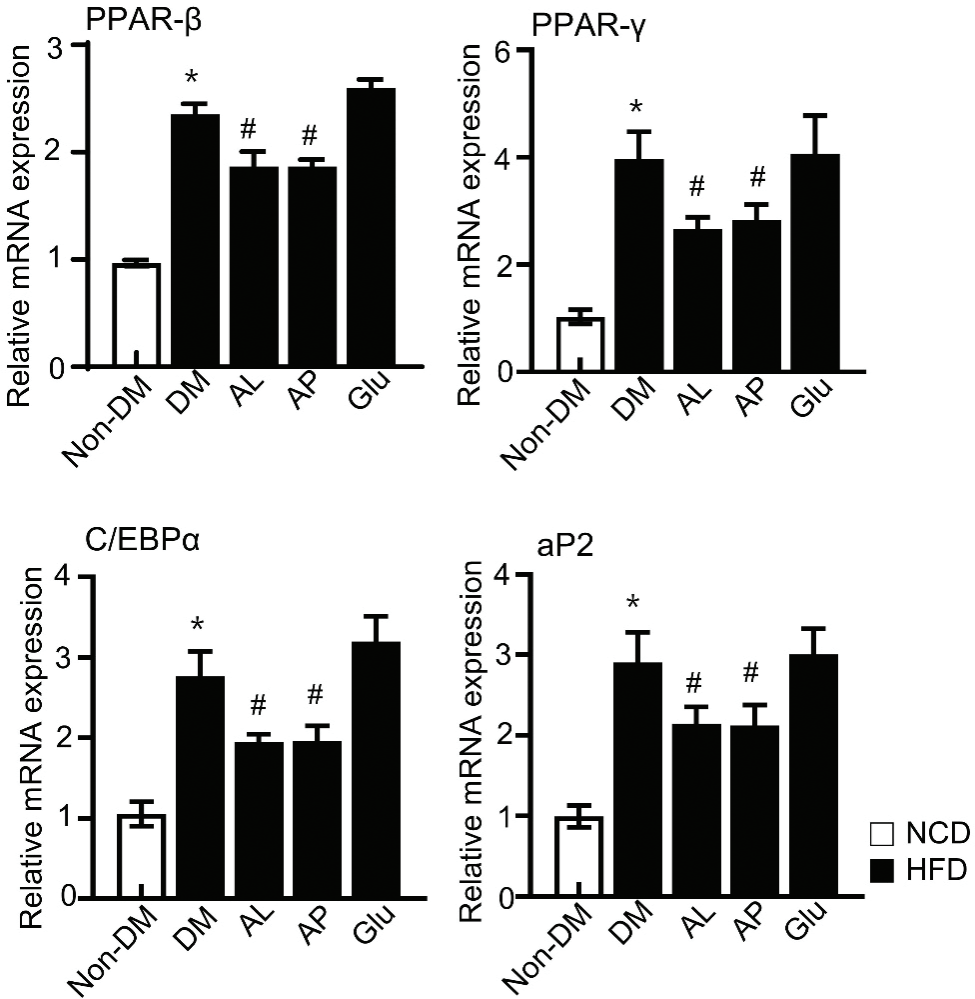
Effect of D-allulose on the gene expressions of the key adipogenic transcription factors. Effect of D-allulose on the gene expressions of the key adipogenic factors, PPARβ, PPARγ, C/EBP α, and aP2. Results are means ± SEM from three separate experiments. (*n* = 3, ^*^P < 0.05 vs. Non-DM group, ^#^*P* < 0.05 vs. DM-group, ^**^P < 0.05 vs. DM-Glu group). Non-DM, undifferentiated group; DM, differentiated group; AP, Powdery D-allulose; AL, Liquid D-allulose; Glu, glucose.

Supplementation of D-allulose during adipocyte differentiation and the mRNA expression level of adipogenesis-related genes such as PPARβ, PPARγ, aP2, and C/EBPα were measured by real-time PCR. As shown in Fig. 3, PPARβ, PPARγ, aP2, and C/EBPα increased in the differentiated adipocytes. In contrast, adipogenesis-related genes, PPARβ, PPARγ, aP2, and C/EBPα were reduced in the AL and AP treated groups.

### Effect of D-allulose on body weight and blood glucose level in HFD-induced adiposity in vivo

**Fig. 4:**
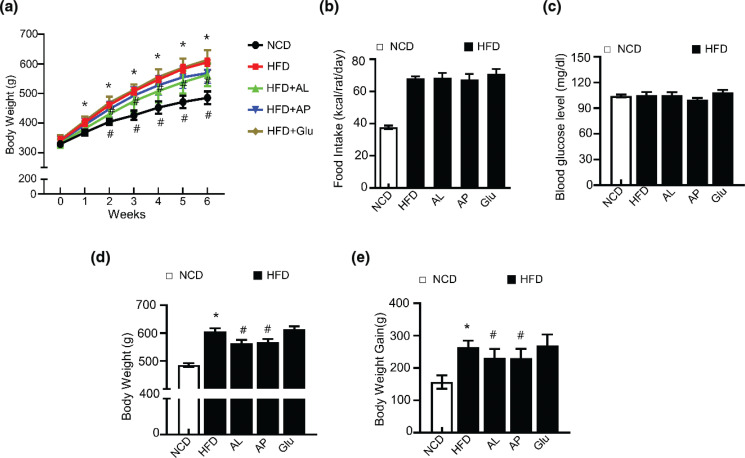
Effects of D-allulose on body weight gain, food intake, and fasting glucose level in high-fat diet-induced obese SD rats. Rats were placed on either an NCD or 60% HFD for 8 weeks and were supplemented with 0.4 g/kg AP, 0.4 g/kg AL, and 0.4 g/kg Glu. (a) Bodyweight and (b) food intake were recorded once a week, (c) blood glucose and (d) body weight were assessed last week. (e) Bodyweight gain was assessed last week. Results are means ± SEM from eight separate experiments (*n* = 8, *P < 0.05 vs. NCD-group, ^#^*P* < 0.05 vs. HFD-group). NCD, Normal chow diet; HFD, high-fat diet; AP, Powdery D-allulose; AL, Liquid D-allulose; Glu, glucose.

To evaluate the anti-adiposity effect of D-allulose on the development of adiposity *in vivo*, 8 weeks’ old rats were subjected to either a normal chow diet (NCD) or an HFD. Body weight was recorded during the experimental period (Fig. 4). At the end of 6 weeks, a significant decrease in body weight was observed in the D-allulose-treated HFD group compared to the HFD group (Fig. 4a, d, e). However, there was no significant difference in dietary intake between the D-allulose-treated HFD group and the HFD-induced group (Fig. 4b). Consistently, the weight gain in AL- and AP-fed HFD rats exhibited a significant reduction compared to the HFD group. Collectively, these observations indicate the efficacy of D-allulose as a dietary supplement in preventing adiposity. We further analyzed serum glucose levels. Glucose levels in the sera of each group did not display significant differences (Fig. 4c).

### D-allulose ameliorates biochemical parameters of blood in HFD-fed rats

First, we examined the serum ALT and AST levels, representing the biochemical index of hepatic injury (Table 1). The observations indicated that D-allulose treatment significantly alleviates serum ALT and AST levels relative to the HFD-fed rats. Additionally, HFD-fed rats showed a significantly higher TG, TC, and LDL-c than the D-allulose-treated HFD group. In contrast, D-allulose supplementation significantly inhibited the increase of TG, TC, and LDL-c relative to HFD-fed rats. Furthermore, the serum leptin content increased significantly in the HFD-fed group and decreased the serum adiponectin content. However, administration of D-allulose effectively reversed the level of adiponectin and leptin (Table 1).

**Table 1:** Effects of D-allulose on the serum levels of biochemicals parameters in high-fat diet-induced SD rats

Biochemicals parameters	NCD	HFD	HFD+AL	HFD+AP	HFD+Glu
ALT (IU/L)	8.81 ± 0.38	42.24 ± 1.08*	26.43 ± 0.78^#^	22.79 ± 0.94^#^	44.10 ± 1.14^#^
AST (IU/L)	8.78 ± 0.36	45.15 ± 1.43*	32.61 ± 0.85^#^	30.27 ± 1.05^#^	43.19 ± 2.29^#^
Triglyceride (mg/dL)	40.47 ± 2.08	67.47 ± 3.42*	49.25 ± 3.17^#^	54.62 ± 3.91^#^	64.30 ± 4.32^#^
Total-cholesterol (mg/dL)	83.51 ± 1.62	106.07 ± 3.71*	76.34 ± 2.46^#^	79.95 ± 3.05^#^	108.53 ± 3.42^#^
LDL-cholesterol (mg/dL)	30.74 ± 1.39	44.54 ± 1.60*	31.00 ± 0.97^#^	27.47 ± 2.06^#^	42.60 ± 1.86^#^
Leptin (ng/mL)	27.29 ± 0.99	39.27 ± 1.54*	32.59 ± 1.72^#^	31.43 ± 1.47^#^	36.45 ± 1.73^#^
Adiponectin (μg/mL)	7.07 ± 0.27	4.10 ± 0.27*	6.19 ± 0.28^#^	6.42 ± 0.18^#^	4.72 ± 0.24^#^

Result are means ± SEM (*n* = 8).

* *P* < 0.05 versus NCD-group

# *P* < 0.05 versus HFD-group.

### Effects of D-allulose on white adipose tissue weight in HFD-induced adiposity

White adipose tissue (WAT) is a hormonally active component orchestrating total fat. Aberrantly, elevated WAT deposits are referred to as visceral adiposity. Hence, abdominal fat, perirenal fat, and epididymal fat weight were measured soon after sacrifice. As shown in Table 2, the weight of HFD-fed rats demonstrated a significant increase in fat weight than NCD-fed rats. In contrast, these WAT masses are significantly ameliorated in AP- and Al-fed rats compared to HFD rats. Also, we observed an apparent decrease in total fat pad mass in AL- and AP-fed rats compared to NCD rats.

**Table 2:** Effects of D-allulose on WAT weight distribution in high-fat diet-induced SD rats. Results are means ± SEM (*n* = 8)

White adipose tissue	NCD	HFD	HFD+AL	HFD+AP	HFD+Glu
Abdominal fat	11.56 ± 0.84	23.35 ± 0.95*	17.45 ± 0.67^#^	18.41 ± 0.93^#^	23.06 ± 1.13
Epididymal fat	7.62 ± 0.65	17.90 ± 0.82*	13.40 ± 0.54^#^	13.54 ± 0.79^#^	18.14 ± 1.17
Perirenal fat	3.57 ± 0.24	6.33 ± 0.45*	5.43 ± 0.38	6.17 ± 0.53	6.25 ± 0.48
Total fat	22.75 ± 1.45	47.57 ± 2.00*	36.28 ± 1.10^#^	38.12 ± 1.89^#^	47.45 ± 2.63

* *P* < 0.05 versus NCD-group

# *P* < 0.05 versus HFD-group

### D-allulose improves adiposity via regulation of adipogenic related genes

**Fig. 5:**
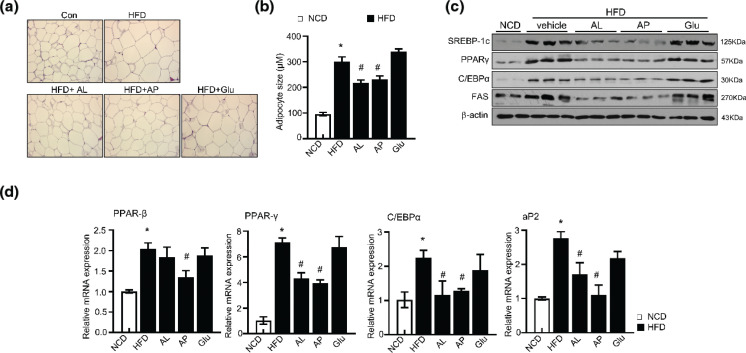
Effects of D-allulose on lipid accumulation and FAS, PPARγ, C/EBPα, and SREBP1 expression in eWAT. (a) The epididymal white adipose tissue (eWAT) from representative rats in each group was fixed, embedded in paraffin, and stained with H&E. Images are shown at the original magnification of 100×. (b) The average diameter of adipocytes in the eWAT of each group. Total protein and RNA were prepared from eWAT, and the protein expression (c) and the mRNA levels of (d) FAS, PPARγ, C/EBPα, and SREBP-1c were analyzed by western blot and quantitative reverse transcription-PCR (qRT-PCR). Results are means ± SEM from three separate experiments (*n* = 8, *P < 0.05 vs. NCD-group, ^#^*P* < 0.05 vs. HFD-group). NCD, Normal chow diet; HFD, high-fat diet; AP, Powdery D-allulose; AL, Liquid D-allulose; Glu, glucose.

Epididymal white adipose tissue (eWAT), a typical VAT (Visceral adipose tissue), is used to study the relationship between adipose tissue and adiposity. To demonstrate whether the amelioration of weight gain and VAT cumulation in AL- and AP-fed rats is implicated in adipose tissue alterations, confirmatory tests were done. Histological H&E staining of eWAT in HFD-fed rats revealed apparent adipocyte hypertrophy in glucose-fed groups compared to the control group (Fig. 5a, b). Contradictorily, the adipocyte size of eWAT is smaller in AL and AP administered groups compared to adipocytes isolated from HFD rats. This observation indicated that hypertrophy could be improved by administering the allulose. Moreover, the expansion and renewal of adipocytes are tightly regulated at the transcriptional level. During differentiation of adipocytes and hypertrophy, PPARγ regulates lipid accumulation (28). To determine the effect of D-allulose on HFD-induced adipocyte differentiation and lipid accumulation, the mRNA and protein expression levels of adipose differentiation-related transcription factors (PPARβ, PPARγ, C/EBPα, SREBP-1c, FAS, and aP2) were measured. D-allulose supplementation significantly inhibited the expression of differentiation-related transcription factors compared to HFD-induced rats (Fig. 5c). Also, relative mRNA expression of PPARγ and C/EBPα was lowered in AL- and AP-fed HFD-induced groups, and other adipogenesis-related genes such as FAS and aP2 decreased in AL and AP treatment groups (Fig. 5d).

### D-allulose promotes deacetylation of PGC-1α in WAT via AMPK-SIRT1 axis

**Fig. 6:**
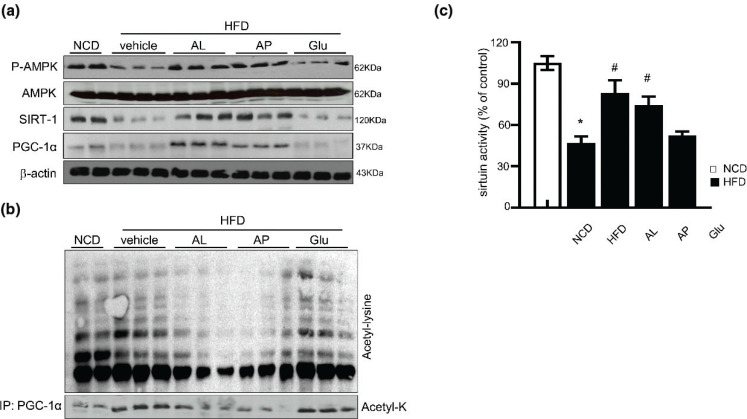
Effect of AMPK and SIRT-1 of D-allulose on deacetylation of PGC1α in WAT. (a) Effects of D-allulose on P-AMPK and SIRT-1 protein levels were analyzed in WAT by WB analysis. (b) Immunoprecipitation of PGC-1α followed by WB for acetyl-lysine from WAT. (c) D-allulose inhibits SIRT1 activity. WAT; white adipose tissue. Results are means ± SEM from three separate experiments (*n* = 3, *P < 0.05 vs. NCD-group, ^#^*P* < 0.05 vs. HFD-group). NCD, Normal chow diet; HFD, high-fat diet; AP, Powdery D-allulose; AL, Liquid D-allulose; Glu, glucose.

In this study, the activities of important nutrient sensors such as AMPK and SIRT1 in adipose tissue were determined. AMPK phosphorylation and SIRT1 expression in HFD-fed rats were reduced in control. However, D-allulose restored AMPK phosphorylation and SIRT1 expression (Fig. 6a). Previous reports show that PGC-1α is activated by deacetylation and interacts with SIRT1, initiating its transcriptional activity. Moreover, Sirt1 activation by AMPK takes place, which has beneficial effects on mitochondrial biogenesis via deacetylation of PGC-1α. Hence, acetylation of PGC-1α was measured under D-allulose treatment. It was observed that D-allulose supplementation decreased the acetylated form of PGC-1α in HFD-fed rats. Together, SIRT-1 activity linked to PGC-1α deacetylation was diminished in the HFD-group compared to the NCD-fed group, whereas administration of D-allulose successfully restored SIRT-1 activity linked PGC-1α deacetylation (Fig. 6c). Thus, we assumed that the effect of D-allulose on PGC-1α activation is mediated by SIRT1 activity.

## Discussion

In this study, D-allulose reduces intracellular fat accumulation in adipocytes, positively correlating with reduced adiposity. D-allulose supplementation attenuated the expression of lipogenesis genes, suggesting the D-allulose’s inhibitory mechanism on lipid accumulation, weight gain, and adipose depositing. Besides, the effect of D-allulose in WAT is correlated to the AMPK-SIRT1-PGC-1α signaling pathway. Here, we demonstrated that AMPK, a major sensor that maintains body energy homeostasis, regulates the expression of genes involved in adipocyte energy metabolism by acting in coordination with SIRT1, another metabolic sensor. Our *in vitro* and *in vivo* studies showed the improved effect of D-allulose on lipogenesis and adipocyte differentiation through metabolic shift controlling fat accumulation. During adipocyte differentiation, advanced stage-expressed transcription factors regulate the differentiation of adipocytes, C/EBPα and PPARγ facilitate lipogenesis-related expression involved in energy metabolism, proliferation, and inflammation of adipocytes (20). PPARγ and C/EBPα act as crucial interactive regulators that mediate organismic metabolism diseases that comprise several aspects of pathologic diseases such as obesity, cancer, and inflammation (29). The repression of PPARγ and C/EBPα owing to the administration of D-allulose is also evident in the present study.

It is well-known that visceral adipose tissue has multiple roles in orchestrating whole-body energy homeostasis. It potentially buffers most of the excess energy by responding to alterations in nutrient intake through adipocyte expansion and adipogenesis (12). The AMPK-SIRT1 axis is a recently established metabolic signal mechanism (16). The SIRT1 is an NAD^+^-dependent deacetylation enzyme (30) that regulates lipid metabolism through its deacetylase activity and directly or indirectly contributing to lipid signaling (31). D-allulose transforms the adipose tissue and reduces the expression of adipocyte lipid synthesis via the AMPK-SIRT1 axis. Besides, the activation of AMPK affects the expressions of lipid synthesis-related genes and a few glycolytic or lipogenic enzymes like SREBP and FAS, acetyl-CoA carboxylase (ACC), and pyruvate kinase (32, 33).

Additionally, the total fatty weight has a marked reduction after treatment with D-allulose. Our *in vitro* and *in vivo* study observations proved these conceptions that D-allulose inhibits adiposity by repressing adipogenic factors and promotes thermogenesis in which AMPK-SIRT1-PGC-1α is involved. This translates the deacetylation of SIRT1 targets such as PGC-1α in response to the pharmacological or physiological AMPK activation (34). The expression levels of p-AMPK and SIRT1 proteins are markedly restored on D-allulose administrated rats compared to HFD-fed rats. Afterward, a predominant increase of Sirt1 activity and deacetylation of PGC-1α was observed in D-allulose treated groups. Similarly, in adipocytes, D-allulose treatment activated the AMPK pathway and its associated SIRT1-PGC-1α activation, indicating that under AMPK, activation represses genes controlled by the fat regulator PPAR-γ, including genes mediating fat storage. Hypertrophy of differentiated adipocytes causes dysfunction, inflammation, hyperglycemia, dyslipidemia, and altered adipokine secretion (35). At the late differentiation stage, the aP2 gene is expressed as preadipocytes are fully differentiated into adipocytes. The aP2 gene is overexpressed only in mature adipocytes and acts like adipokine, which regulates systemic metabolism, but is significantly reduced with D-allulose treatment. This proves that D-allulose treatment effectively inhibits the post-differentiation process in 3T3-L1 cells. Adipocytokine is a cytokine secreted only from adipose tissue, and leptin and adiponectin are the representative cytokines. It is known that leptin is involved in suppressing food intake and stimulation of thermogenesis, whereas adiponectin is considered to be a pleiotropic organ-protective protein, which is produced only by adipose tissue, and is reduced with the progress of obesity (36). Besides, AMPK regulates hormones, such as leptin and adiponectin, which are associated with appetite and metabolism (37). This study established that D-allulose effectively lowers the level of leptin in the serum and increases the level of adiponectin, which dramatically improves hepatic fatty acid oxidation.

Scientists and people’s attention is naturally diverted to adiposity as people’s food and physical habits frequently changed. Lack of exercise and excess energy intake increased the prevalence of adiposity in modern life. Therefore, growing studies have showed their concentration to develop natural products with fewer side effects (38). Here, we have shown that D-allulose administration had a weight-reducing effect in a food intake-independent manner. This is contradictory with previously published literature on the short-term effect of D-allulose administration. However, our data are consistent with cumulative food intake with D-allulose (39). Few related studies determined that D-allulose will promote GLP-1 secretion and modulate appetite, similar to some GLP-1R agonists. This demonstrated a temporary decrease in food intake and then consequently observed a recovery on the level of food intake in chronic administration (40, 41). In addition, it was confirmed that when D-allulose was administered, the adipose ratio in the tissue of the obese rats was induced by the HFD and the size of the epididymal adipocyte tissue was reduced. Therefore, it was confirmed that D-allulose has an apparent efficacy in reducing adipose tissue.

## Conclusion

In summary, this study reports that D-allulose supplementation inhibited lipid and triglycerides accumulation in HFD-fed rats and differentiation cell model *in vitro*. At the same time, D-allulose decreased the expression of adipogenesis gene, preventing fat accumulation in WAT in obese rats. In addition, D-allulose activates AMPK/SIRT1/PGC-1α signals in adipose tissue to prevent adiposity and inhibit adipocyte differentiation in adipose tissue. Our study provides evidence that D-allulose supplements effectively reduced HFD-induced adiposity by regulating metabolism in WAT. Thus, D-allulose could be used as a nutraceutical to prevent adiposity but further investigations are necessary for complete drug design.

## Ethics statement

All experimental animals used for this study were cared for in accordance with the regulation of the Care and Use of Laboratory Animals Guide of Jeonbuk National University with approval from the Institutional Animal Care and Use Committee of Jeonbuk National University Hospital (cuh-IACUC-2019-09). Animal studies were performed under the guidelines and regulations set and approved by IACUC, Jeonbuk National University Hospital.

## Conflict of interest and funding

The authors declare no conflict of interest. The authors have not received any funding or benefits from industry or elsewhere to conduct this study.

## Supplementary Material

D-allulose ameliorates adiposity through the AMPK-SIRT1-PGC-1α pathway in HFD-induced SD rats

## References

[1] de Melo KM, de Oliveira FTB, Costa Silva RA, Gomes Quindere AL, Marinho Filho JDB, Araujo AJ, et al. α, β-Amyrin, a pentacyclic triterpenoid from Protium heptaphyllum suppresses adipocyte differentiation accompanied by down regulation of PPARγ and C/EBPα in 3T3-L1 cells. Biomed Pharmacother 2019; 109: 1860–6. doi: 10.1016/j.biopha.2018.11.027 30551441

[2] Liu P, Hsieh P, Lin H, Liu T, Wu H, Chen C, et al. Grail is involved in adipocyte differentiation and diet-induced obesity. Cell Death Dis 2018; 9(5): 525. doi: 10.1038/s41419-018-0596-8 29743578 PMC5943410

[3] Salmenniemi U, Ruotsalainen E, Pihlajamaki J, Vauhkonen I, Kainulainen S, Punnonen K, et al. Multiple abnormalities in glucose and energy metabolism and coordinated changes in levels of adiponectin, cytokines, and adhesion molecules in subjects with metabolic syndrome. Circulation 2004; 110(25): 3842–8. doi: 10.1161/01.CIR.0000150391.38660.9B 15596567

[4] Li J, Papadopoulos V. Translocator protein (18 kDa) as a pharmacological target in adipocytes to regulate glucose homeostasis. Biochem Pharmacol 2015; 97(1): 99–110. doi: 10.1016/j.bcp.2015.06.020 26123521

[5] Xiao P, Yang Z, Sun J, Tian J, Chang Z, Li X, et al. Silymarin inhibits adipogenesis in the adipocytes in grass carp Ctenopharyngodon idellus *in vitro* and *in vivo*. Fish Physiol Biochem 2017; 43(6): 1487–500. doi: 10.1007/s10695-017-0387-7 28646459

[6] Oshima H, Kimura I, Izumori K. Psicose contents in various food products and its origin. Food Sci Technol Res 2006; 12: 137–43. doi: 10.3136/fstr.12.137

[7] Zhang W, Yu S, Zhang T, Jiang B, Mu W. Recent advances in d-allulose: physiological functionalities, applications, and biological production. Trends Food Sci Technol 2016; 54: 127–37. doi: 10.1016/j.tifs.2016.06.004

[8] Kishida K, Martinez G, Iida T, Yamada T, Ferraris RP, Toyoda Y. D-allulose is a substrate of glucose transporter type 5 (GLUT5) in the small intestine. Food Chem 2019; 277: 604–8. doi: 10.1016/j.foodchem.2018.11.003 30502192

[9] Van Opstal AM, Hafkemeijer A, van den Berg-Huysmans AA, Hoeksma M, Mulder TPJ, Pijl H, et al. Brain activity and connectivity changes in response to nutritive natural sugars, non-nutritive natural sugar replacements and artificial sweeteners. Nutr Neurosci 2021; 24(5): 395–405. doi: 10.1080/1028415X.2019.1639306 31288630

[10] Han Y, Park H, Choi BR, Ji Y, Kwon EY, Choi MS. Alteration of microbiome profile by d-allulose in amelioration of high-fat-diet-induced obesity in mice. Nutrients 2020; 12(2): 352. 10.3390/nu12020352 PMC7071329 32013116

[11] Pratchayasakul W, Jinawong K, Pongkan W, Jaiwongkam T, Arunsak B, Chunchai T, et al. Not only metformin, but also D-allulose, alleviates metabolic disturbance and cognitive decline in prediabetic rats. Nutr Neurosci 2020; 5: 1–13. 10.1080/1028415X.2020.1840050 33151133

[12] Maeng HJ, Yoon JH, Chun KH, Kim ST, Jang DJ, Park JE, et al. Metabolic stability of d-allulose in biorelevant media and hepatocytes: comparison with fructose and erythritol. Foods 2019; 8(10): 448. doi: 10.3390/foods8100448 PMC6835332 31581594

[13] Kimura T, Kanasaki A, Hayashi N, Yamada T, Iida T, Nagata Y, et al. D-allulose enhances postprandial fat oxidation in healthy humans. Nutrition 2017; 43–44: 16–20. doi: 10.1016/j.nut.2017.06.007 28935140

[14] Shintani T, Yamada T, Hayashi N, Iida T, Nagata Y, Ozaki N, et al. Rare sugar syrup containing d-allulose but not high-fructose corn syrup maintains glucose tolerance and insulin sensitivity partly via hepatic glucokinase translocation in wistar rats. J Agric Food Chem 2017; 65(13): 2888–94. doi: 10.1021/acs.jafc.6b05627 28209058

[15] Kanasaki A, Niibo M, Iida T. Effect of d-allulose feeding on the hepatic metabolomics profile in male Wistar rats. Food Funct 2021; 12: 3931–3938. doi: 10.1039/D0FO03024D 33977954

[16] Canto C, Gerhart-Hines Z, Feige JN, Lagouge M, Noriega L, Milne JC, et al. AMPK regulates energy expenditure by modulating NAD+ metabolism and SIRT1 activity. Nature 2009; 458(7241): 1056–60. doi: 10.1038/nature07813 19262508 PMC3616311

[17] Hardie DG. AMP-activated/SNF1 protein kinases: conserved guardians of cellular energy. Nat Rev Mol Cell Biol 2007; 8(10): 774–85. doi: 10.1038/nrm2249 17712357

[18] Gerhart-Hines Z, Rodgers JT, Bare O, Lerin C, Kim SH, Mostoslavsky R, et al. Metabolic control of muscle mitochondrial function and fatty acid oxidation through SIRT1/PGC-1α. EMBO J 2007; 26(7): 1913–23. doi: 10.1038/sj.emboj.7601633 17347648 PMC1847661

[19] Ghaben AL, Scherer PE. Adipogenesis and metabolic health. Nat Rev Mol Cell Biol 2019; 20(4): 242–58. doi: 10.1038/s41580-018-0093-z 30610207

[20] Farmer SR. Transcriptional control of adipocyte formation. Cell Metab 2006; 4(4): 263–73. doi: 10.1016/j.cmet.2006.07.001 17011499 PMC1958996

[21] Rosen ED, MacDougald OA. Adipocyte differentiation from the inside out. Nat Rev Mol Cell Biol 2006; 7(12): 885–96. doi: 10.1038/nrm2066 17139329

[22] Ali AT, Hochfeld WE, Myburgh R, Pepper MS. Adipocyte and adipogenesis. Eur J Cell Biol 2013; 92(6–7): 229–36. doi: 10.1016/j.ejcb.2013.06.001 23876739

[23] Khalilpourfarshbafi M, Gholami K, Murugan DD, Abdul Sattar MZ, Abdullah NA. Differential effects of dietary flavonoids on adipogenesis. Eur J Nutr 2019; 58(1): 5–25. doi: 10.1007/s00394-018-1663-8 29541908 PMC6424933

[24] Evans RM, Barish GD, Wang YX. PPARs and the complex journey to obesity. Nat Med 2004; 10(4): 355–61. doi: 10.1038/nm1025 15057233

[25] Garcia D, Shaw RJ. AMPK: mechanisms of cellular energy sensing and restoration of metabolic balance. Mol Cell 2017; 66(6): 789–800. doi: 10.1016/j.molcel.2017.05.032 28622524 PMC5553560

[26] Jones JR, Barrick C, Kim KA, Lindner J, Blondeau B, Fujimoto Y, et al. Deletion of PPARγ in adipose tissues of mice protects against high fat diet-induced obesity and insulin resistance. Proc Natl Acad Sci U S A 2005; 102(17): 6207–12. doi: 10.1073/pnas.0306743102 15833818 PMC556131

[27] Madsen MS, Siersbaek R, Boergesen M, Nielsen R, Mandrup S. Peroxisome proliferator-activated receptor gamma and C/EBPalpha synergistically activate key metabolic adipocyte genes by assisted loading. Mol Cell Biol 2014; 34(6): 939–54. doi: 10.1128/MCB.01344-13 24379442 PMC3958030

[28] Macdougall CE, Wood EG, Loschko J, Scagliotti V, Cassidy FC, Robinson ME, et al. Visceral adipose tissue immune homeostasis is regulated by the crosstalk between adipocytes and dendritic cell subsets. Cell Metab 2018; 27(3): 588–601.e4. doi: 10.1016/j.cmet.2018.02.007 29514067 PMC5846800

[29] Vitale SG, Lagana AS, Nigro A, La Rosa VL, Rossetti P, Rapisarda AM, et al. Peroxisome proliferator-activated receptor modulation during metabolic diseases and cancers: master and minions. PPAR Res 2016; 2016: 6517313. doi: 10.1155/2016/6517313 28115924 PMC5225385

[30] Chang HC, Guarente L. SIRT1 and other sirtuins in metabolism. Trends Endocrinol Metab 2014; 25(3): 138–45. doi: 10.1016/j.tem.2013.12.001 24388149 PMC3943707

[31] Wang YM, Huang TL, Meng C, Zhang J, Fang NY. SIRT1 deacetylates mitochondrial trifunctional enzyme alpha subunit to inhibit ubiquitylation and decrease insulin resistance. Cell Death Dis 2020; 11(10): 821. doi: 10.1038/s41419-020-03012-9 33009367 PMC7532168

[32] Lee MS, Han HJ, Han SY, Kim IY, Chae S, Lee CS, et al. Loss of the E3 ubiquitin ligase MKRN1 represses diet-induced metabolic syndrome through AMPK activation. Nat Commun 2018; 9(1): 3404. doi: 10.1038/s41467-018-05721-4 30143610 PMC6109074

[33] Long YC, Zierath JR. AMP-activated protein kinase signaling in metabolic regulation. J Clin Invest 2006; 116(7): 1776–83. doi: 10.1172/JCI29044 16823475 PMC1483147

[34] Nisoli E, Clementi E, Paolucci C, Cozzi V, Tonello C, Sciorati C, et al. Mitochondrial biogenesis in mammals: the role of endogenous nitric oxide. Science 2003; 299(5608): 896–9. doi: 10.1126/science.1079368 12574632

[35] den Besten G, Bleeker A, Gerding A, van Eunen K, Havinga R, van Dijk TH, et al. Short-chain fatty acids protect against high-fat diet-induced obesity via a PPARγ-dependent switch from lipogenesis to fat oxidation. Diabetes 2015; 64(7): 2398–408. doi: 10.2337/db14-1213 25695945

[36] Combs TP, Berg AH, Obici S, Scherer PE, Rossetti L. Endogenous glucose production is inhibited by the adipose-derived protein Acrp30. J Clin Invest 2001; 108(12): 1875–81. doi: 10.1172/JCI14120 11748271 PMC209474

[37] Huynh MK, Kinyua AW, Yang DJ, Kim KW. Hypothalamic AMPK as a regulator of energy homeostasis. Neural Plast 2016; 2016: 2754078. doi: 10.1155/2016/2754078 27547453 PMC4980534

[38] Cho YR, Lee JA, Kim YY, Kang JS, Lee JH, Ahn EK. Anti-obesity effects of Clausena excavata in high-fat diet-induced obese mice. Biomed Pharmacother 2018; 99: 253–60. doi: 10.1016/j.biopha.2018.01.069 29334669

[39] Iwasaki Y, Sendo M, Dezaki K, Hira T, Sato T, Nakata M, et al. GLP-1 release and vagal afferent activation mediate the beneficial metabolic and chronotherapeutic effects of D-allulose. Nat Commun 2018; 9(1): 113. doi: 10.1038/s41467-017-02488-y 29317623 PMC5760716

[40] Xu F, Lin B, Zheng X, Chen Z, Cao H, Xu H, et al. GLP-1 receptor agonist promotes brown remodelling in mouse white adipose tissue through SIRT1. Diabetologia 2016; 59(5): 1059–69. doi: 10.1007/s00125-016-3896-5 26924394

[41] van Bloemendaal L, RG IJ, Ten Kulve JS, Barkhof F, Konrad RJ, Drent ML, et al. GLP-1 receptor activation modulates appetite- and reward-related brain areas in humans. Diabetes 2014; 63(12): 4186–96. doi: 10.2337/db14-0849 25071023

