# Supplementary material for: D-allulose ameliorates adiposity through the AMPK-SIRT1-PGC-1α pathway in HFD-induced SD rats
Source: Food Nutr Res. 2021 Dec 21;65:10.29219/fnr.v65.7803. doi: 10.29219/fnr.v65.7803 (PMC8829832; doi:10.29219/fnr.v65.7803)
Supplement: D-allulose ameliorates adiposity through the AMPK-SIRT1-PGC-1α pathway in HFD-induced SD rats [file FNR-65-7803-s001.docx]

**Figures and Legends**


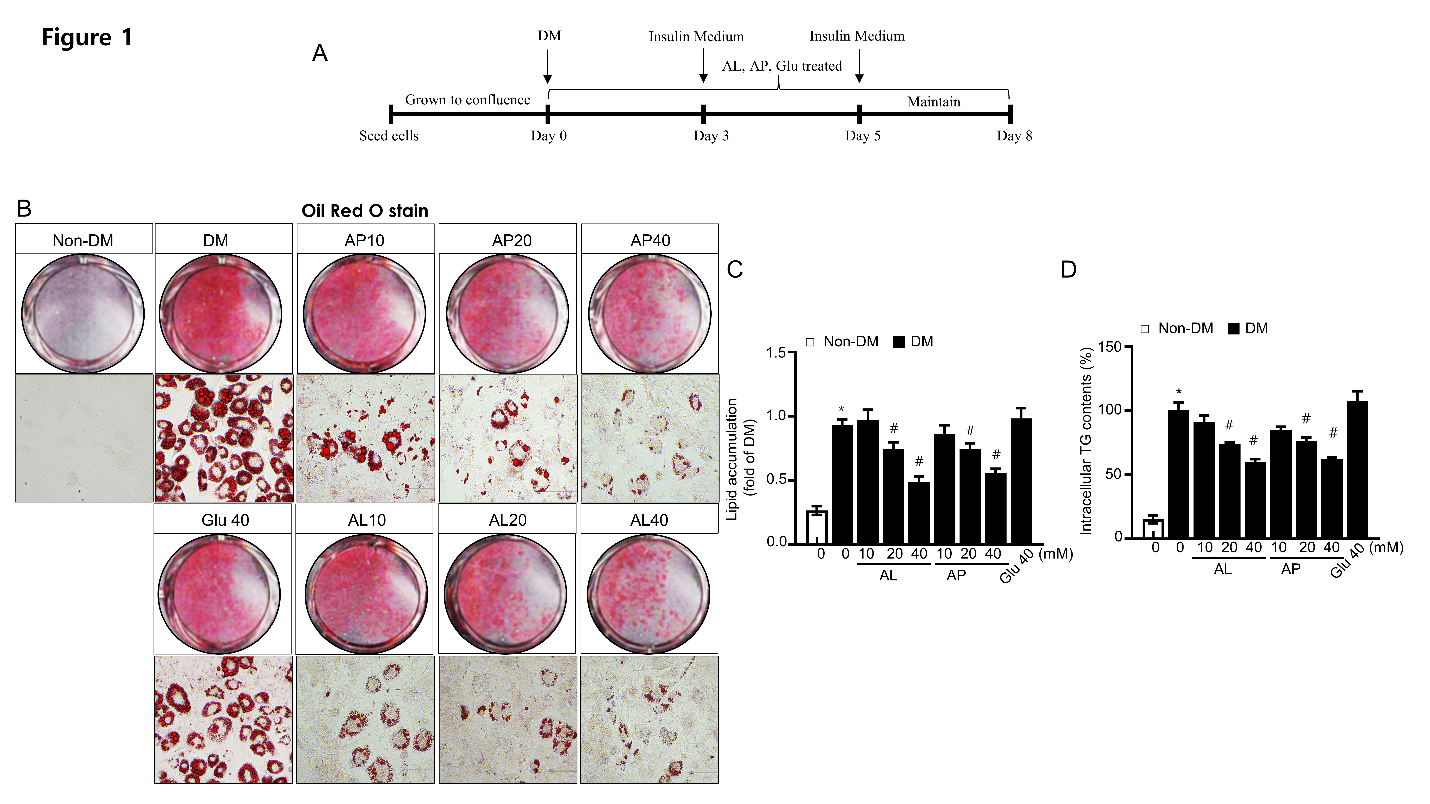


**Fig. 1. Anti-adipognic effects of D-allulose in 3T3-L1 adipocytes.** (A) Schematic diagram of the differentiation protocol for 3T3-L1 differentiation. (B-C) Effects of D-allulose on the differentiation of 3T3-L1 cells. 3T3-L1 cells were cultured in adipocyte differentiation cocktail media with or without the treatment of D-allulose. After 7 days of culture, the cells were stained with Oil Red O and then photographed under the microscope (magnification 40x). Measurement of relative lipid content in different treatment groups. 3T3-L1 cells were cultured in adipogenic cocktail media with or without the D-allulose treatment (Right). (D) Effect of D-allulose on the triglyceride deposition in differentiated 3T3-L1 cells.3T3-L1 cells were cultured in adipocyte differentiation cocktail media with or without the treatment of D-allulose. Results are means ± SEM from 3 separate experiments. (n = 3, **P*< 0.05 vs. Non-DM group, ^#^*P*< 0.05 vs. DM group). DM, differentiated medium; Non-DM group, undifferentiated group; DM group, differentiated group; AP, Powdery D-allulose; AL, Liquid D-allulose; Glu, glucose.


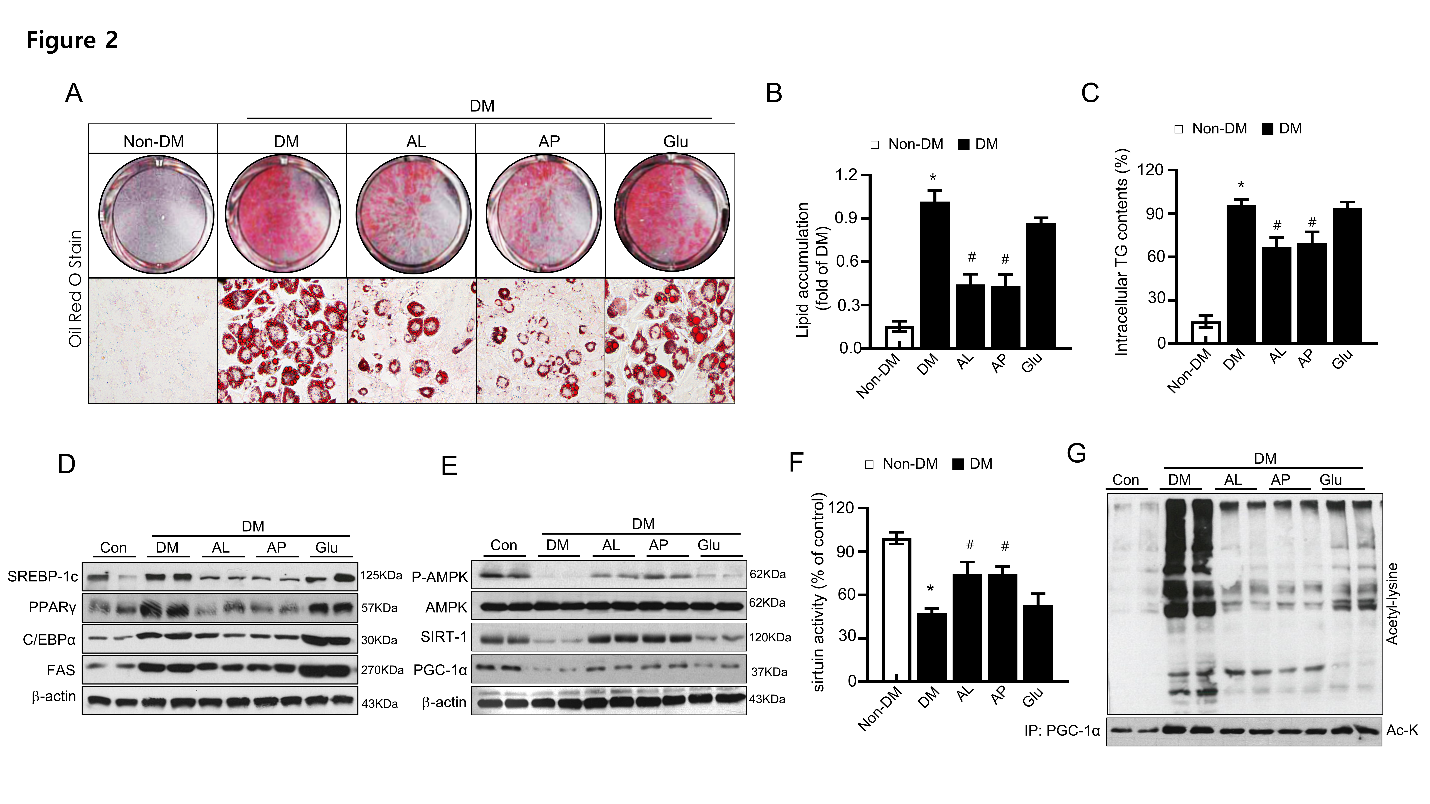


**Fig. 2. Effect of AMPK and SIRT-1 of D-allulose on promotes deacetylation of PGC1α in 3T3-L1 adipocytes.** Effects of D-allulose on the differentiation of 3T3-L1 cells. (A) Post confluent 3T3‐L1 cells were differentiated in the absence or presence of 20mM AL and 20mM AP for 8 days. Lipid droplets were measured by Oil Red O staining. (B) Lipid content was quantified by measuring absorbance. (C) Intracellular TG in D-allulose‐treated 3T3-L1 cells was measured. (D) Expression of adipogenesis factors SREBP-1c, PPAR-γ, C/EBPα, and FAS protein levels were analyzed in 3T3-L1 cells by WB analysis. (E) Effects of D-allulose on P-AMPK, SIRT-1, and PGC-1α protein levels were analyzed in 3T3-L1 cells by WB analysis. (F) Sirtuin activity. (G) Immunoprecipitation of PGC-1α followed by WB for acetyl-lysine from differentiated 3T3-L1 cells. Results are means ± SEM from 3 separate experiments. (n = 3, **P*< 0.05 vs. Non-DM group, ^#^ *P*< 0.05 vs. DM-group, ^**^*P*< 0.05 vs. DM-Glu group). Non-DM, undifferentiated group; DM, differentiated group; AP, Powdery D-allulose; AL, Liquid D-allulose; Glu, glucose.


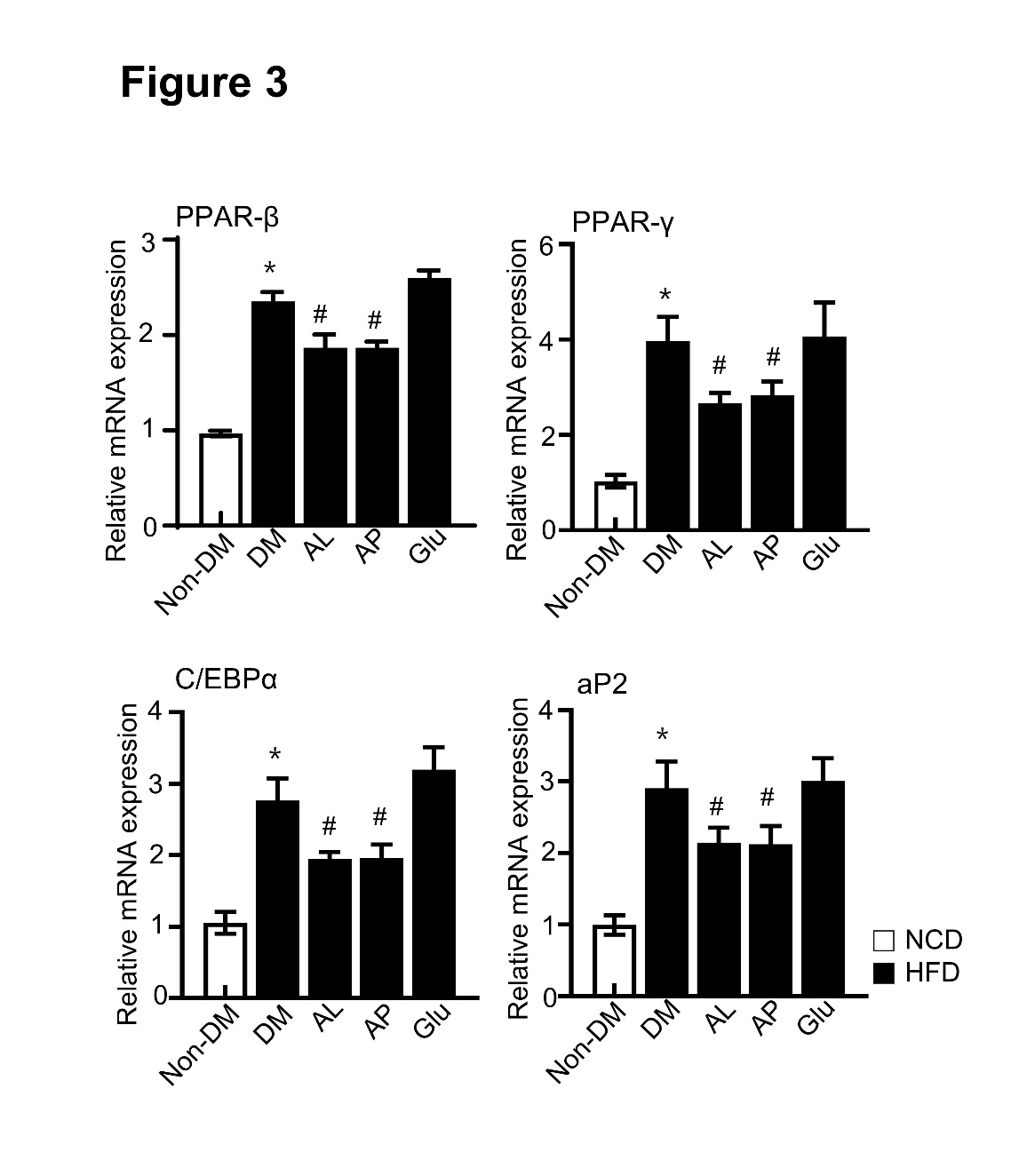


**Fig. 3. Effect of D-allulose on the gene expressions of the key adipogenic transcription factors.** Effect of D-allulose on the gene expressions of the key adipogenic factors, PPARβ, PPARγ, C/EBP α and aP2. Results are means ± SEM from 3 separate experiments. (n = 3, ^*^*P*< 0.05 vs. Non-DM group, ^#^*P* < 0.05 vs. DM-group, ^**^*P*< 0.05 vs. DM-Glu group). Non-DM, undifferentiated group; DM, differentiated group; AP, Powdery D-allulose; AL, Liquid D-allulose; Glu, glucose.


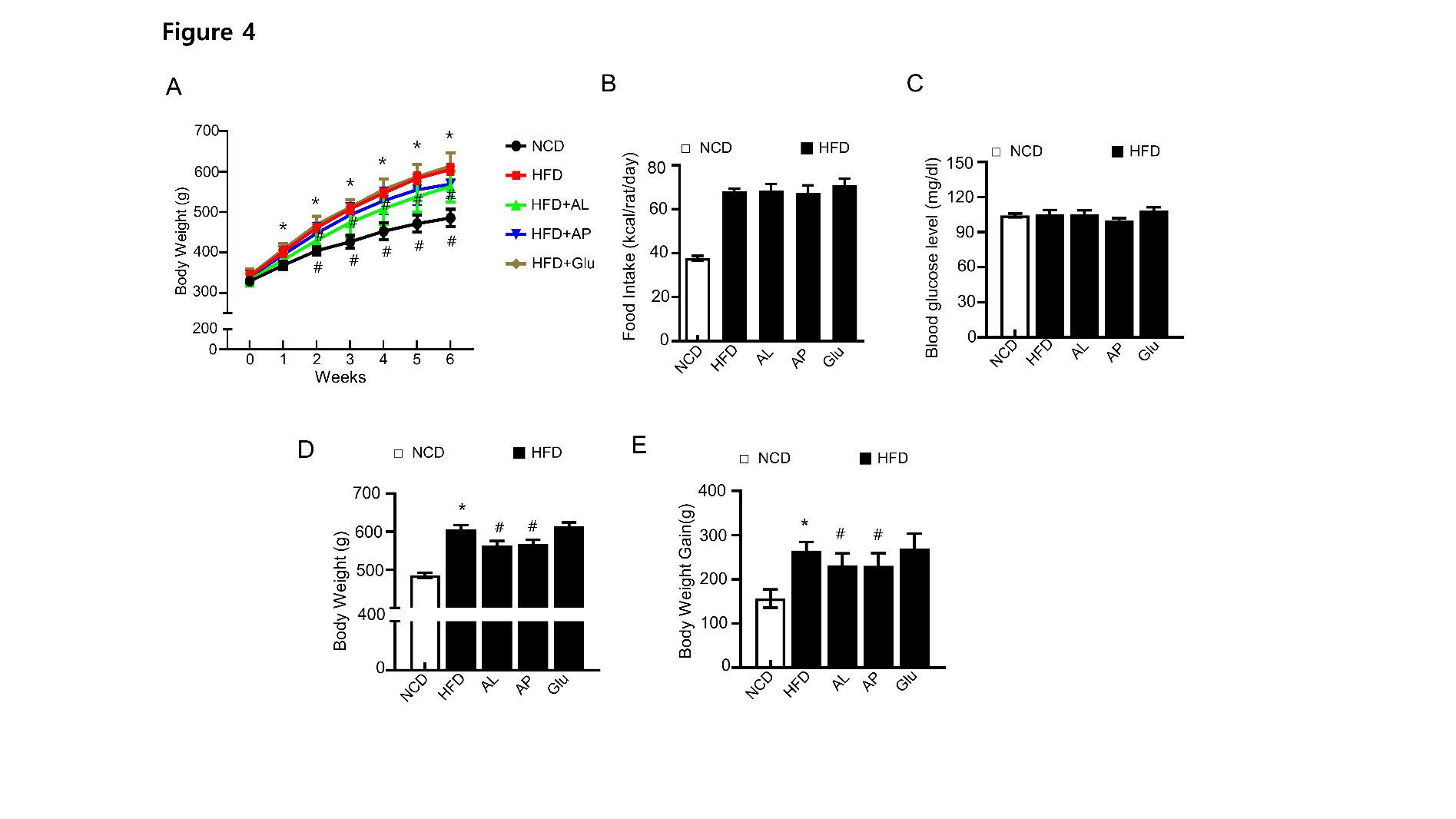


**Fig. 4. Effects of D-allulose on body weight gain, food intake and fasting glucose level in high-fat diet-induced obese SD rats.** Rats were placed on either an NCD or 60% HFD for eight weeks and were supplemented with 0.4g/kg AP, 0.4g/kg AL and 0.4g/kg Glu. (A) Bodyweight, (B) food intake was recorded once a week, (C) blood glucose and (D) body weight was assessed last week. (E) Bodyweight gain was assessed last week. Results are means ± SEM from 8 separate experiments (n = 8, **P*< 0.05 vs. NCD-group, ^#^*P*< 0.05 vs. HFD-group). NCD, Normal chow diet; HFD, high-fat diet; AP, Powdery D-allulose; AL, Liquid D-allulose; Glu, glucose.


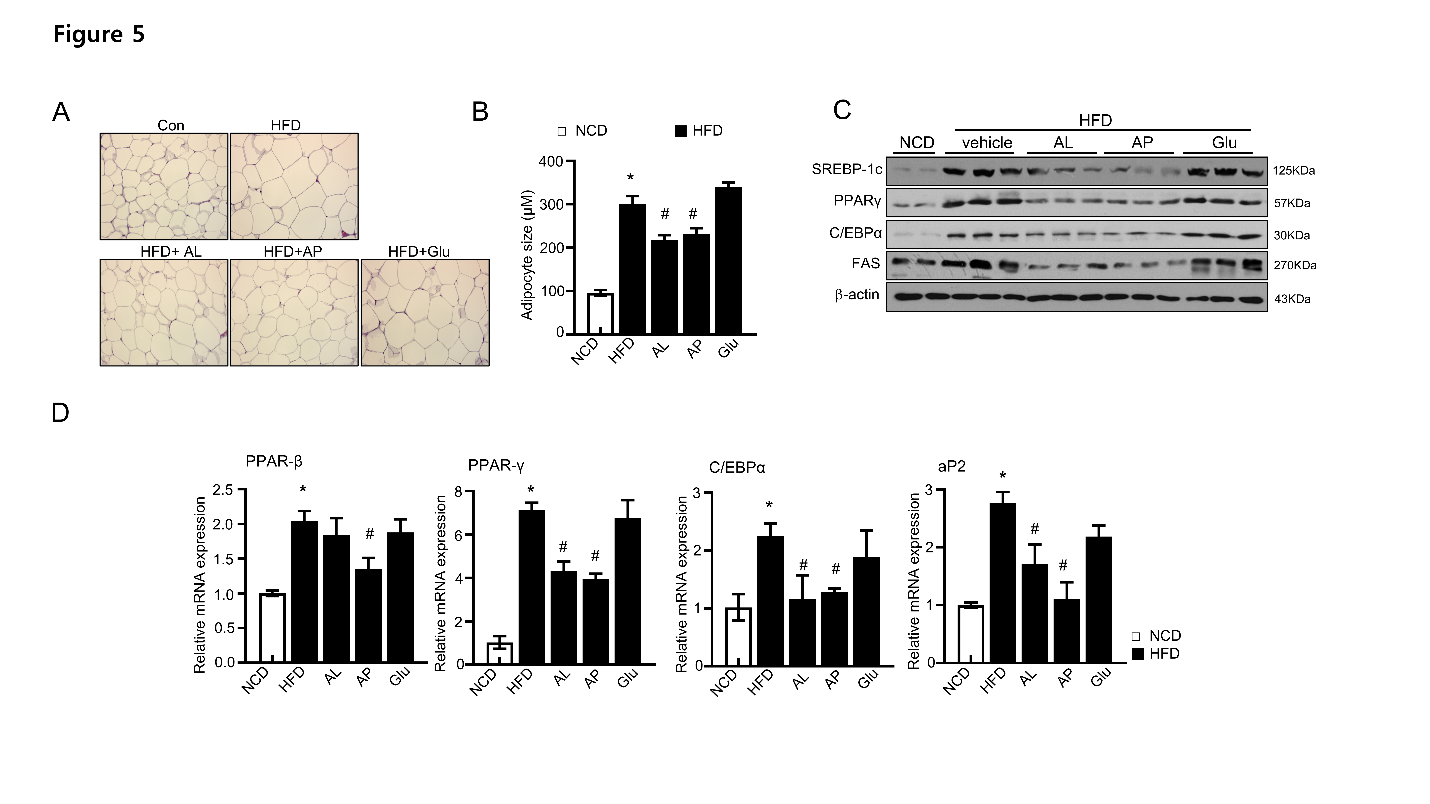


**Fig. 5. Effects of D-allulose on lipid accumulation and FAS, PPARγ, C/EBPα, and SREBP1 expression in eWAT.** (A) The epididymal white adipose tissue (eWAT) from representative rats in each group was fixed, embedded in paraffin, and stained with H&E. Images are shown at the original magnification of 100x. (B) The average diameter of adipocytes in the eWAT of each group. Total protein and RNA were prepared from eWAT, and the protein expression (C) and the mRNA levels of (D) FAS, PPARγ, C/EBPα, and SREBP-1c were analyzed by western blot and quantitative RT-PCR (qRT-PCR). Results are means ± SEM from 3 separate experiments (n = 8, **P*< 0.05 vs. NCD-group, ^#^*P*< 0.05 vs. HFD-group). NCD, Normal chow diet; HFD, high-fat diet; AP, Powdery D-allulose; AL, Liquid D-allulose; Glu, glucose.


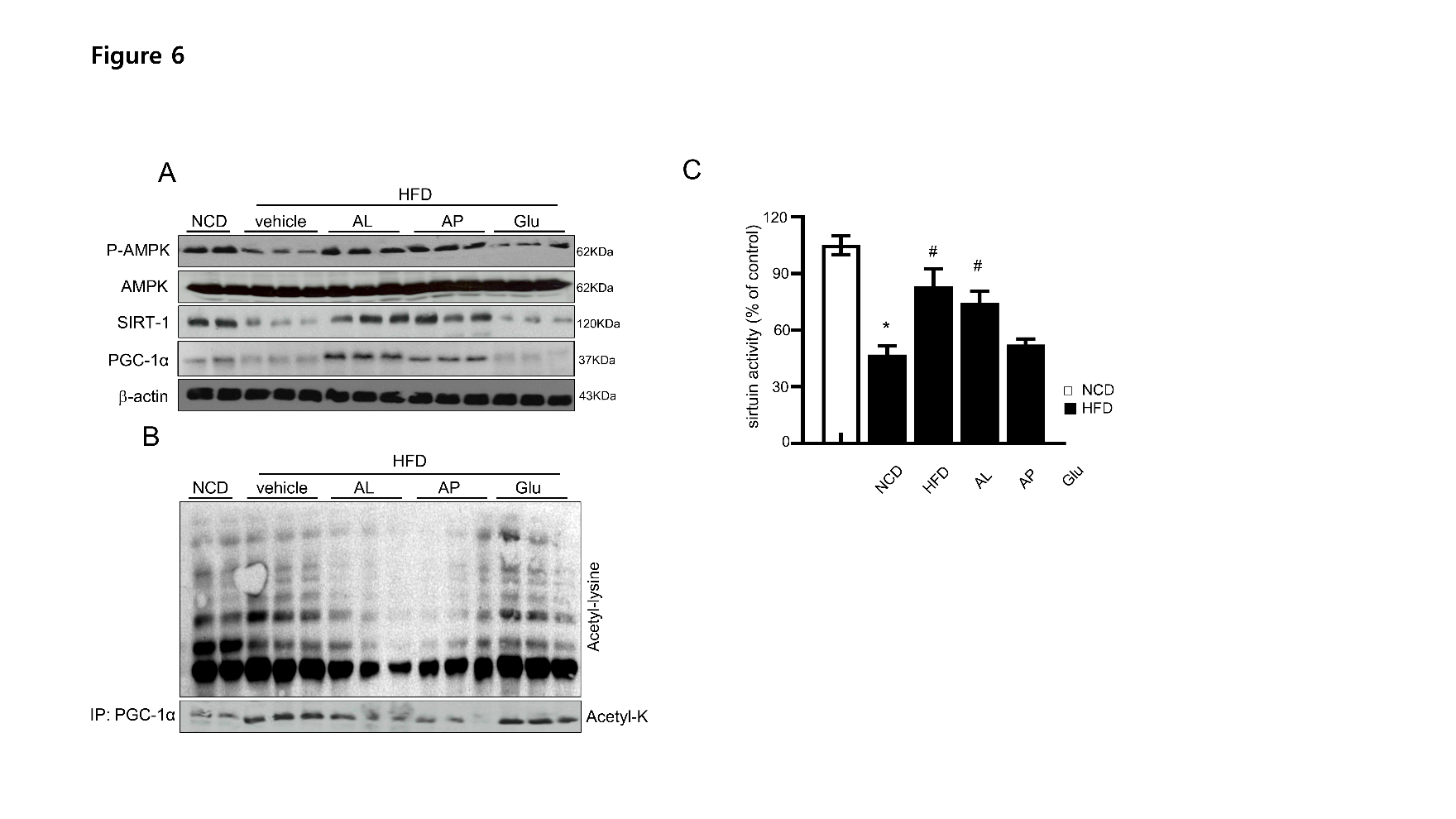


**Fig. 6. Effect of AMPK and SIRT-1 of D-allulose on promotes deacetylation of PGC1α in WAT.** (A) Effects of D-allulose on P-AMPK and SIRT-1 protein levels were analyzed in WAT by WB analysis. (B) Immunoprecipitation of PGC-1α followed by WB for acetyl-lysine from WAT. (C) D-allulose inhibits SIRT1 activity. WAT; white adipose tissue. Results are means ± SEM from 3 separate experiments (n = 3, **P* < 0.05 vs. NCD-group, ^#^*P* < 0.05 vs. HFD-group). NCD, Normal chow diet; HFD, high-fat diet; AP, Powdery D-allulose; AL, Liquid D-allulose; Glu, glucose.

**Table 1.** Effects of D-allulose on the serum levels of biochemicals parameters in high-fat diet-induced SD rats. Result are means ± SEM(n=8).

|  | **NCD** | **HFD** | **HFD+AL** | **HFD+AP** | **HFD+Glu** |
| --- | --- | --- | --- | --- | --- |
| **ALT(IU/L)** | 8.81±0.38 | 42.24±1.08* | 26.43±0.78^#^ | 22.79±0.94^#^ | 44.10±1.14^#^ |
| **AST(IU/L)** | 8.78±0.36 | 45.15±1.43* | 32.61±0.85^#^ | 30.27±1.05^#^ | 43.19±2.29^#^ |
| **Triglyceride(mg/dl)** | 40.47±2.08 | 67.47±3.42* | 49.25±3.17^#^ | 54.62±3.91^#^ | 64.30±4.32^#^ |
| **Total-Cholesterol(mg/dl)** | 83.51±1.62 | 106.07±3.71* | 76.34±2.46^#^ | 79.95±3.05^#^ | 108.53±3.42^#^ |
| **LDL-Cholesterol(mg/dl)** | 30.74±1.39 | 44.54±1.60* | 31.00±0.97^#^ | 27.47±2.06^#^ | 42.60±1.86^#^ |
| **Leptin(ng/ml)** | 27.29±0.99 | 39.27±1.54* | 32.59±1.72^#^ | 31.43±1.47^#^ | 36.45±1.73^#^ |
| **Adiponectin(μg/ml)** | 7.07±0.27 | 4.10±0.27* | 6.19±0.28^#^ | 6.42±0.18^#^ | 4.72±0.24^#^ |

**P* < 0.05 vs. NCD-group, ^#^*P* < 0.05 vs. HFD-group). NCD, Normal chow diet; HFD, high-fat diet; AP, Powdery D-allulose; AL, Liquid D-allulose; Glu, glucose.

**Table 2.** Effects of D-allulose on WAT weight distribution in high-fat diet-induced SD rats. Result are means ± SEM(n=8).

|  | **NCD** | **HFD** | **HFD+AL** | **HFD+AP** | **HFD+Glu** |
| --- | --- | --- | --- | --- | --- |
| **Abdominal fat** | 11.56±0.84 | 23.35±0.95* | 17.45±0.67^#^ | 18.41±0.93^#^ | 23.06±1.13 |
| **Epididymal fat** | 7.62±0.65 | 17.90±0.82* | 13.40±0.54^#^ | 13.54±0.79^#^ | 18.14±1.17 |
| **Perirenal fat** | 3.57±0.24 | 6.33±0.45* | 5.43±0.38 | 6.17±0.53 | 6.25±0.48 |
| **Total fat** | 22.75±1.45 | 47.57±2.00* | 36.28±1.10^#^ | 38.12±1.89^#^ | 47.45±2.63 |

**P* < 0.05 vs. NCD-group, ^#^*P* < 0.05 vs. HFD-group). NCD, Normal chow diet; HFD, high-fat diet; AP, Powdery D-allulose; AL, Liquid D-allulose; Glu, glucose.

**Supplementary Information**

Supplementary Table 1. Real-time PCR primer sequences.

| **Gene** | **Sense (5’-3’)** | **Antisense (5’-3’)** |
| --- | --- | --- |
| **PPARγ** | GGTGAAACTCTGGGAGATCCTCC | AGCAACCATTGGGTCAGCTCT |
| **PPARβ** | GAGGACAAACCCACGGTAAA | GGCCACTTCCTCTTTCTCCT |
| **CEBPα** | AGTCGGTGGATAAGAACAGCAACG | GCTGTTTGGCTTTATCTCGGCTC |
| **aP2** | GCGTGGAATTCGATGAAA TCA | CCCGCCATCTAGGGTTATGA |
| **β-actin** | CCTAAGGCCAACCGTAAAG | GGTCCACATTCTTTTCCTGATACTG |
